# In Vitro Effect of the Length of Relocated Cervical Margin with Casting Post and Core, Prefabricated Fiber Post, and Polyethylene Fiber with a Composite Core on Fracture Resistance and Marginal Integrity

**DOI:** 10.1155/2024/9274141

**Published:** 2024-08-27

**Authors:** Naghmeh Musapoor, Hamid Neshandar Asli, Soroosh Mokhtari, Yasamin Babaee Hemmati, Mehran Falahchai

**Affiliations:** ^1^ Department of Prosthodontics Dental Sciences Research Center School of Dentistry Qazvin University of Medical Sciences, Qazvin, Iran; ^2^ Department of Prosthodontics Dental Sciences Research Center School of Dentistry Guilan University of Medical Sciences, Rasht, Iran; ^3^ Department of Prosthodontics Dental Sciences Research Center School of Dentistry Arak University of Medical Sciences, Arak, Iran; ^4^ Department of Orthodontics Dental Sciences Research Center School of Dentistry Guilan University of Medical Sciences, Rasht, Iran

## Abstract

**Objective:**

This study aimed to assess the effect of length of the relocated cervical margin with casting post and core (CP), prefabricated fiber post and composite core (PFP), and polyethylene fiber-reinforced composite (PEFRC) on fracture resistance and marginal integrity.

**Materials and Methods:**

In this in vitro study, 70 sound human maxillary premolars were divided into seven groups according to the type of post and core system and length of the relocated cervical margin (*n* = 10): control (no preparation), PFP-3, PEFRC-3, CP-3 with 3 mm of cervical margin relocation (CMR), PFP-6, PEFRC-6, and CP-6 (with 6 mm of CMR). The samples were restored with zirconia crowns (except the control group). Epoxy resin replicas were fabricated before and after thermomechanical loading. Marginal integrity was assessed at the luting cement-core, core-tooth, and luting cement-enamel interfaces under a scanning electron microscope (SEM) (×200). Fracture resistance and failure mode were subsequently assessed. Data were analyzed by independent *t*-test, paired *t*-test, ANOVA, Tukey–Games Howell, Mann–Whitney, Kruskal–Wallis, Wilcoxon, Mann–Whitney with Bonferroni correction, and Fisher–Freeman–Halton tests (*α* = 0.05).

**Results:**

The marginal integrity of the groups with 3 mm of CMR followed the following order: PEFRC > PFP > CP at all interfaces (*P*  < 0.05). In 6-mm CMR groups, this order was CP < PFP = PEFRC at the luting cement-core and (CP < PEFRC) = PFP at the core-tooth interface. No significant difference was found in fracture resistance when comparing the 3-mm CMR groups with each other (*P*  > 0.05). PFP-6 showed higher FR than CP-6 (*P*  < 0.001). PEFRC-6 had no significant difference with PFP-6 and CP-6 (*P*  > 0.05). In each post and core system, 3-mm CMR groups showed higher marginal integrity and fracture resistance (*P*  < 0.05).

**Conclusion:**

Increasing the length of the relocated cervical margin decreased the marginal integrity and fracture resistance of all three systems of CP, PFP, and PEFRC.

## 1. Introduction

Cervical margin relocation (CMR) was first introduced by Dietschi and Spreafico in 1998 and is also known as the proximal box elevation and deep margin elevation [1, 2]. In this approach, the subgingival margin in deep cervical lesions is relocated supragingivally by using restorative materials within one treatment session. Accordingly, a supragingival margin is created, enabling more precise conventional and digital impression making, easier oral hygiene practice, and easier access for elimination of excess cement and lowering the risk of inflammation and periodontal disease [2, 3, 4].

Maxillary premolars are subjected to high occlusal forces due to their position in dental arch [5]. The reduction in fracture resistance of endodontically treated teeth due to removal of tooth structure can be as high as 63% when both the mesial and distal marginal ridges are removed [6, 7]. Considering all the above, selection of a proper restorative procedure is highly important to guarantee a long-term clinical service [8].

Intracanal posts are commonly used to provide adequate retention for the core supporting the restoration and also to enhance the biomechanical properties and fracture resistance of teeth with extensive coronal destruction particularly premolars due to small anatomical size of the crown [5, 9, 10]. Casting post (CP) and core systems were long regarded as the gold standard for tooth reconstruction [10]. Introduction of prefabricated fiber posts (PFP) enabled benefitting from the advantages of adhesive bonding to tooth structure and mechanical properties closer to those of dentin [11]. It has been discussed that proper adherence to the protocols of the contemporary adhesive dentistry can prevent the increase in polymerization shrinkage and subsequent reduction in bond strength, and a unform interface may be achieved between the restorative material and sound tooth structure as such [12]. Accordingly, fiber-reinforced composite was proposed as intracanal post for endodontically treated teeth along with a composite core to serve as an alternative to metal posts and PFP [13]. The advantages of these posts include higher adaptation to root canal compared with prefabricated posts and high flexibility and having a modulus of elasticity close to that of dentin structure [13].

Several previous studies assessed the marginal adaptation of indirect restorations (inlays, onlays, and full crowns) and showed that the application of CMR approach did not decrease the marginal adaptation [3, 14, 15, 16]. However, some other studies reported lower marginal adaptation in this approach, compared with the placement of finish line and restoration margin directly on dentin [17, 18, 19]. Moreover, some previous studies [3, 18, 20] demonstrated that the application of the CMR approach, irrespective of restoration type (inlay, only, and endocrown) and restoration material, had no adverse effect on fracture resistance [21].

In the clinical setting, deep cavities with variable depths may be encountered depending on the amount of lost tooth structure due to caries, old restorations, or fracture. In previous studies, the buccolingual width of the proximal cavity which was altered by the CMR technique was between 3 and 5 mm [18, 22, 23]. Nonetheless, conclusive evidence is lacking regarding the acceptable length of margin for supragingival relocation in the CMR approach or the effect of cavity dimensions on fracture resistance and marginal adaptation of restorations in this approach. Thus, the purpose of this in vitro study was to assess the effect of length of the relocated cervical margin with CP, PFP, and polyethylene fiber-reinforced composite (PEFRC) on fracture resistance and marginal integrity. The null hypothesis was that the length of the relocated cervical margin and the type of post and core system would have no significant effect on fracture resistance and marginal integrity.

## 2. Materials and Methods

Sample size estimation was performed by PASS 11 by using one-way ANOVA sample size calculation formula according to a previous study by Elbanna et al. [24], considering a statistical power of 0.85, an error rate of 0.05, and a standard deviation of 10.90. The minimum sample size for this in vitro study was calculated to be 10. Seventy sound human maxillary premolars with equal dimensions (9–10 mm buccolingual width, 7–8 mm mesiodistal width, and 14–16 mm root length) [7] were included in this study. The teeth had been extracted for orthodontic treatment or due to periodontal problems. The inclusion criteria were sound teeth with no caries or fracture line, completely formed roots, and presence of two distinct canals without severe curvature or large width extending from the pulp chamber to the apex (Vertucci's type Ⅳ) [25] as confirmed radiographically. The root trunk (from the cementoenamel junction (CEJ) to the mesial or distal groove) was measured to be 7–8 mm by a vernier digital caliper [26]. Moreover, all specimens were inspected under a stereomicroscope (SZX9; Olympus optical Co. Ltd., Tokyo, Japan). The teeth were stored in 0.1% thymol solution [7]. The root surface was coated with a thin 0.3-mm layer of light-body polyvinyl siloxane impression material (Panasil; Kettenbach GmbH & Co. KG, Eschenburg, Germany) for simulation of the periodontal ligament. The teeth were mounted in autopolymerizing acrylic resin (Demotec 20, Demotec Siegfried Demel, Nidderau, Germany) to 2 mm below their CEJ [7, 24]. The teeth were subsequently assigned to seven groups (*n* = 10) according to the type of post and core system and length of the relocated cervical margin (3 and 6 mm) (Table 1):

  (I) Control
  (II) Prefabricated fiber post and composite core (PFP-3)
  (III) Polyethylene fiber-reinforced composite (PEFRC-3)
  (IV) Casting post and core (CP-3)
  (V) PFP-6
  (VI) PEFRC-6
  (VII) CP-6

Ten teeth were assigned to the control group and received no preparation. In the remaining 60 teeth, a putty index (Panasil; Kettenbach GmbH & Co. KG, Eschenburg, Germany) was obtained prior to preparation. After preparation of a standard access cavity with a cylindrical diamond bur (bur No. S6836KR, Komet, Lemgo, Germany) which included complete deroofing of the pulpal chamber and achieving a straight-line access to the first curvature or the apical part of the canal, the root canals were instrumented with ProTaper Next (Dentsply Maillefer, Ballaigues, Switzerland) to size 30 ISO and obturated with gutta-percha (Gutta Percha, DiaDent, Burnaby, British Columbia) and AH Plus epoxy resin sealer (Dentsply, Konstanz, Germany) by the lateral compaction technique [7]. The palatal canal was subsequently emptied to 10 mm below the orifice by using a rotary instrument (Largo drill #2, Angelus, Parana, Brazil) [5, 7].

**Figure 1:**
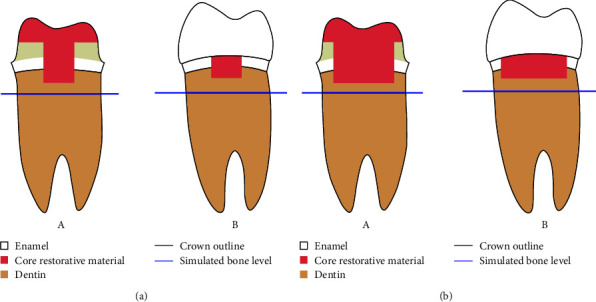
Specimen preparation from the distal view, (a) in the absence of crown and (b) in the presence of crown: (A) 3 mm CMR and (B) 6 mm CMR.

To simulate crown destruction, the test group teeth were decoronated at 3 mm above their CEJ by using a diamond disk (ref no. 983.104.008, Komet, Lemgo, Germany) and high-speed handpiece [7]. The specimens were then assigned to two groups based on their degree of destruction. In 30 teeth, the distal wall with 3-mm marginal length at the midpoint of the buccolingual dimension was relocated to 1 mm below the CEJ by using a cylindrical diamond bur (bur No. S6836KR, Komet, Lemgo, Germany). In the remaining 30 teeth, the length of the relocated margin was 6 mm. Next, the teeth in each group were reconstructed with three different post and core systems, namely, PFP, PEFRC, and CP. After core fabrication, 1 mm of axial space and 2 mm of occlusal space were considered in all test specimens for fabrication of a full crown. A chamfer finish line was created with a round-end conical green belt diamond bur (bur No. S6856, Komet, Lemgo, Germany) 1 mm above the CEJ (located in the enamel all around the tooth and in the core in part of the distal surface) [7] (Figure 1).

In the PFP groups, the root canals were treated with Clearfil SE Bond primer (Clearfil SE Bond, Kuraray Noritake Dental, Tokyo, Japan) according to the manufacturer's instructions, and it was light-polymerized with a halogen curing-unit (Ptilux 501, Kerr, CT, USA). The fiber posts (Reforpost #2, Angelus, Parana, Brazil) were cleaned with 70% alcohol, and silane (Silano Angelus, Angelus, Parana, Brazil) was applied on each fiber post for 1 min. Dual-cure resin cement (RelyX Unicem; 3M ESPE, Seefeld, Germany) was applied for cementation of the posts according to the manufacturer's instructions. The posts were then cut at 3 mm above the orifice. Core buildup was performed by using Clearfil SE Bond and dual-cure composite resin (Core Flo DC LITE, Bisco, IL, USA) by application of 2-mm-thick increments. Each layer was light-polymerized for 40 s [7].

In the PEFRC groups, airborne particle abrasion was first performed with a microsandblaster (Dento-Prep, Ronvig, Daugaard, Denmark) by using 50 *µ*m aluminum oxide particles (2.5 bar pressure, 2 cm distance, and 10 s) perpendicular to the surface [7]. After rinsing the specimens with saline water, immediate dentin sealing was performed for all dentin surfaces except in axial areas in contact with the crown by using Clearfil SE Bond [27]. Next, 26 mm of a polyethylene fiber (Ribbond thin, Ribbond, WA, USA) was cut and dipped in wetting resin (Ribbond wetting resin, Ribbond, WA, USA). After applying RelyX cement into the canal and on the fibers, the fibers were packed and adapted to the canal walls with an endodontic plugger such that 3 mm of fiber was preserved for retention of composite in the coronal region [7]. The cement was light-polymerized from the occlusal surface for 20 s after the removal of excess cement [28]. After 5 min, one layer of flowable composite (Tetric Flow, Ivoclar Vivadent, Bendererstrasse, Liechtenstein) with 0.5 mm thickness was applied on the cavity surface and light-polymerized for 40 s. Thirty minutes after completion of polymerization, core buildup was performed with composite resin (Light-Core, Bisco, IL, USA) in 1-mm increments [29]. Each layer was light-polymerized for 40 s. One layer of air-blocking barrier (K-Y Johnson & Johnson, New Brunswick, NJ, USA) was finally applied, and the entire core was light-polymerized for 10 s [7, 27, 29].

In the CP groups, one-piece post and core resin patterns were fabricated by using autopolymerizing acrylic resin (GC Corporation, Tokyo, Japan). They were then flasked and cast by using nickel chromium alloy (Wiron 99, Bego, Bremen, Germany). The one-piece cast post and core were then cemented with RelyX cement [30].

The test specimens were scanned with an optical scanner (3 Shape D750,3 Shape, Copenhagen, Denmark), and a full crown was designed (CAD Molder Builder; 3Shape, Copenhagen, Denmark) and milled from presintered monolithic zirconia blocks (Zolid Fx multilayer; Amann Girrbach AG, Koblach, Austria) by using a milling machine (Ceramill; Amann Girrbach AG, Koblach, Austria). The cement thickness was 25 *µ*m in marginal areas and 40 *µ*m in other areas [7]. The sintering time for zirconia blocks was 12 hr at 1,500°C. The fabricated crowns were cemented on their respective samples with RelyX cement and subjected to 50 N load application for 10 min in a device designed for crown cementation to simulate finger pressure [31].

For thermomechanical loading, the specimens were placed in the holder of a chewing simulator (Chewing Simulator 4, SD Mechatronik, Westerham, Germany). A stainless steel ball with 8 mm diameter was used as an antagonist. After application of 50 N mechanical load (1.6 Hz, 1.2 Mio.Cycle, 0.2 s time), 6,000 thermal cycles were applied using 5° and 55°C water baths with a dwell time of 2 min (corresponding to 5 years of clinical service) [3, 7].

**Figure 2:**
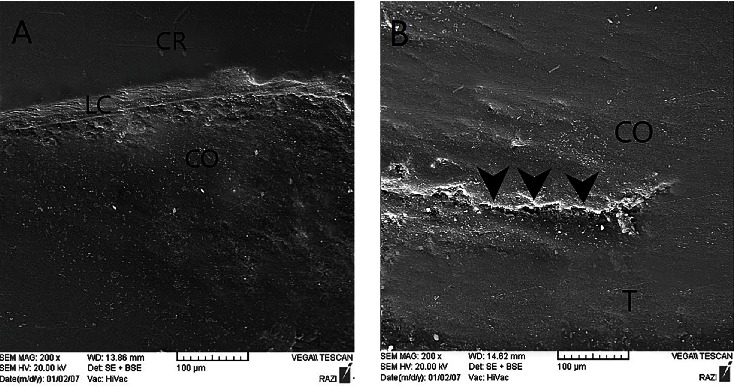
SEM micrographs of epoxy resin replica in the CP-3 group at ×200 magnification: (A) crown-luting cement and luting cement-core interfaces and (B) core-tooth interface. Areas marked on (B) indicate gaps at the respective margins.

To assess the marginal integrity of specimens by using a scanning electron microscope (SEM), impressions were made from the specimens by using polyvinyl siloxane impression material (Panasil; Kettenbach GmbH & Co KG, Eschenburg, Germany) before and after thermomechanical loading to fabricate epoxy resin replicas (Stycast 1266, Emerson & Cuming, Westerlo, Belgium). They were gold sputter-coated, and the marginal integrity in the distal (with the margin located in restorative material) and mesial (with the margin located in enamel) surfaces was quantitively assessed by an experienced operator blinded to the group allocation of the specimens at ×200 magnification (Figure 2). Marginal integrity was evaluated at the following interfaces: (I) crown-luting cement, (II) luting cement-core buildup material at the distal surface (luting cement-core), (III) core buildup material and tooth structure at the distal surface (core-tooth), and (IV) luting cement-enamel (mesial surface of 10 randomly selected specimens) as the control interface. The margins were then assigned to one of the following three groups according to their quality: (I) continuous (no gap), (II) noncontinuous (presence of gaps along the margin), and (III) undetectable. Finally, the marginal integrity was calculated as the percentage ratio of continuous margin to all margin types [14].

**Figure 3:**
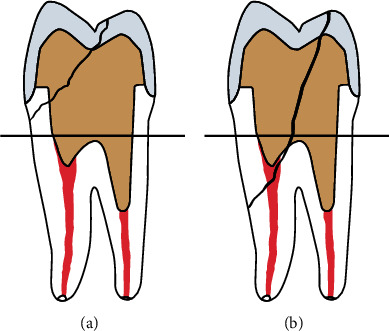
(a) Favorable fractures which were defined as repairable fractures in tooth or restoration that occurred above the bone level. (b) Unfavorable fractures that occurred below the bone level.

To measure the fracture resistance of specimens, they were stored at 37°C for 72 hr in the dark [18] and were then transferred to a universal testing machine (Z050: Zwick, Ulm, Germany). They were subjected to compressive load applied to their central fossa by a steel ball with 6 mm diameter at a crosshead speed of 0.5 mm/min parallel to the longitudinal axis of the tooth. To ensure uniform load application, an aluminum foil with 0.5 mm thickness was used at the crown steel ball interface. The load causing fracture was recorded [7]. The mode of failure was categorized as favorable fractures which were defined as repairable fractures in tooth or restoration that occurred above the bone level and unfavorable fractures that occurred below the bone level [7, 20] (Figure 3).

Normal distribution of data was evaluated by the Shapiro–Wilk test. The homogeneity of the variances was analyzed by the Levene test. Given that the assumptions were met, quantitative data were compared by independent *t*-test, paired samples test, ANOVA (with Tukey post hoc test), and Games–Howell test. Given that the assumptions were not met, comparisons were made by the Mann–Whitney test, Wilcoxon signed rank test, and the Kruskal–Wallis test with pairwise comparisons with Mann–Whitney test and Bonferroni correction. The Fisher–Freeman–Halton exact test was used to compare the failure modes among the groups. All statistical analyses were carried out using SPSS version 28 (IBM SPSS Statistics, v28; IBM Corp, NY, USA) at 0.05 level of significance.

## 3. Results

### 3.1. Crown-Luting Cement Interface

Assessment of the crown-luting cement interface in CP-6 group and core-tooth interface in PFP-6 group by the Wilcoxon signed rank test and also other interfaces by the paired samples test revealed that thermomechanical loading significantly decreased the marginal integrity (*P*  < 0.05).

Assessment of the crown-luting cement interface (Table 2) revealed no significant difference among the study groups in marginal integrity neither before (*P*=0.382) nor after (*P*=0.999) thermomechanical loading.

### 3.2. Luting Cement-Core Interface

Assessment of the luting cement-core interface revealed that when different post and core systems were compared, and also in comparison with the control interface, the difference in marginal integrity was significant in each of the 3 mm and 6 mm CMR groups (Table 3) both before and after thermomechanical loading (*P*  < 0.001). Table 2 presents the within-group comparisons.

### 3.3. Core-Tooth Interface

Assessment of the core-tooth interface in 3-mm and 6-mm CMR groups (Table 2) revealed significant differences between the groups both before and after thermomechanical loading (*P*  < 0.001). Table 3 presents the within-group comparisons.

### 3.4. Comparison of Interfaces

Comparison of the luting cement-core, core-tooth, and luting cement-enamel (control) interfaces (Table 2) in each post and core system revealed significant differences among the groups (*P*  < 0.05). Table 3 presents the within-group comparison of the interfaces.

### 3.5. Comparison of 3-mm and 6-mm CMR Groups

At the luting cement-core interface, no significant difference was found in 3-mm and 6-mm CMR groups with PFP (*P*=0.471) and PEFRC (*P*=0.132) systems before thermomechanical loading. The 3-mm CMR groups showed significantly higher marginal integrity than the 6-mm CMR groups in use of CP prior to thermomechanical loading and in all post and core systems after thermomechanical loading (*P*  < 0.05). At the core-tooth interface, the 3-mm CMR groups showed significantly higher marginal integrity than the 6-mm CMR groups in use of CP and PEFRC systems after thermomechanical loading (*P* < 0.001). In use of PFP, no significant difference was found between 3-mm and 6-mm CMR groups before thermomechanical loading (*P*=0.197). However, 3-mm CMR showed significantly higher marginal integrity than the 6-mm CMR groups after thermomechanical loading (*P*  < 0.001).

### 3.6. Fracture Resistance (Table 4)

No significant difference was found when 3-mm CMR groups were compared with each other and also with the control group (*P*=0.614). However, the difference in fracture resistance was significant when the 6-mm CMR groups were compared with each other and also with the control group (*P*  < 0.001). All 6-mm CMR groups showed lower fracture resistance than the control group (*P*  < 0.001). Also, the fracture resistance of PFP group was higher than that of CP group (*P*  < 0.001). Comparison of 3-mm and 6-mm CMR groups revealed significant differences in all types of post and core systems (*P*  < 0.001). In all three post and core systems, 6-mm CMR groups showed lower fracture resistance than the 3-mm CMR groups (*P*  < 0.05).

### 3.7. Assessment of Failure Mode

A significant difference was noted among the groups in this regard (*P*=0.013). The results showed the highest frequency of favorable failures in the control, PFP-3, and PEFRC-6 (100%) groups and the highest frequency of unfavorable failures in the CP-3 group (55.6%). In CMR groups with the same length of relocated margin, PFP and PEFRC had higher frequency of favorable fractures than CP. In the same post and core systems, the 6-mm CMR groups showed higher frequency of favorable fractures than the 3-mm CMR groups (Table 5).

## 4. Discussion

According to the present results, the null hypothesis of the study regarding no significant effect of length of the relocated margin and type of post and core system on marginal integrity, fracture resistance, and failure mode was totally rejected.

To the best of the authors' knowledge, the minimum clinically acceptable marginal integrity has not been mentioned in the literature. In the present study, the marginal integrity ranged from 84.93% to 85.28% at the crown-luting cement interface and 50.39%–88.44% at the luting cement-core and core-tooth interfaces after thermomechanical loading. Previous studies [32, 33] reported the percentage of marginal integrity to range from 83.16% to 100% at the crown-luting cement interface and 61%–92% at the luting cement-core and core-tooth interfaces, which were within the ranges reported in the present study.

Considering the fact that mechanical and thermal stresses generated by thermomechanical loading directly cause mechanical destruction of the bond at the adhesive interface, the reduction in marginal integrity at all interfaces observed in the present study is justifiable [34]. Similar results were reported in previous studies [19, 33]. However, unlike the present study, Ilgnestein et al. [14] assessed the effect of CMR in endodontically treated molar teeth reconstructed with glass ceramic onlay and reported no significant reduction in marginal integrity after thermomechanical loading. Increasing the frequency of thermal cycles in the thermocycling process can increase the microleakage in the adhesive structure [35]. Moreover, unlike glass ceramic restorations, achieving a strong reliable bond to adhesive materials is more difficult in zirconia restorations since they cannot be etched [36]. Thus, this conflict in the results can be due to the differences in restoration material and lower frequency of thermal cycles in the study by Ilgnestein et al. [14], compared with the present study.

The CP group showed the lowest marginal integrity at the luting cement-core interface in both 3- and 6-mm CMR approaches. The reason may be the lower adhesion of alloy than composite resin to resin cement and dental substrate [37]. Higher microleakage of CP, compared with PFP/composite core, was reported in a study by Jung et al. [37], who compared the microleakage at the interface of different post and core systems and tooth structure after dynamic loading.

In the PEFRC group, the marginal integrity at the core-tooth interface was comparable to that at the control interface (luting cement-enamel) in the 3-mm CMR approach. Considering the applied techniques for enhancement of bond strength, improved marginal adaptation and lower microleakage in this group can be explained [29, 38, 39, 40]. No significant difference was found in marginal integrity of luting cement-core and core-tooth interfaces in use of PFP. However, in application of PEFPC in the 3-mm CMR groups, the core-tooth interface showed higher marginal integrity than the luting cement-core interface. This finding can be attributed to the application of bond strength enhancement techniques at the core-tooth interface compared with the luting cement-core interface. The PEFRC group demonstrated higher marginal integrity than the PFP group at the luting cement-core and core-tooth interfaces in the 3-mm CMR approach. However, this difference was not significant in the 6-mm CMR approach. It may be concluded that by increasing the composite volume in the 6-mm CMR, the bond strength enhancement techniques are not as effective as in the 3-mm CMR.

Comparison of the 3-mm and 6-mm CMR groups with the same post and core system after thermomechanical loading revealed lower marginal integrity in the 6-mm CMR groups. Similar results were reported by Elbanna et al. [24], who observed that doubling the length of the relocated margin from 4 mm to 8 mm in the CMR approach significantly increased the microleakage of premolars reconstructed with zirconia full crowns. Thus, the reduction in structural adaptation and marginal integrity in the 6-mm CMR groups can be explained by an increase in polymerization shrinkage due to increased volume of composite resin (in PEFRC and PFP), increased volume of resin pattern, and decreased accuracy of the laboratory procedures (in CP).

The fracture resistance ranged from 1481.95 to 3073.85 N in the present study. The fracture resistance ranged from 1,083 to 1,446 N in a study conducted on premolars reconstructed with ceramic onlay and CMR [18]. The fracture resistance was 1600–1700 N in the study by Ilgenstein et al. [14], on endodontically treated molars reconstructed with ceramic onlay and CMR. Intraorally, the occlusal load ranges from 220 to 540 N at the premolar region [10]. Accordingly, it may be concluded that the adopted reconstruction protocol in all test groups of the present study was clinically acceptable with respect to fracture resistance.

The 3-mm CMR groups had no significant difference in fracture resistance with each other or with the control group. Previous studies [3, 7, 18, 20, 21] demonstrated that application of CMR with 2–3 mm length and 2–3 mm depth below the CEJ had no significant effect on fracture resistance and mechanical properties of reconstructed premolar and molar teeth, irrespective of indirect restoration design (inlay, onlay, and full crown) and restoration material (indirect composite or ceramic). Nonetheless, the fracture resistance in all 6-mm CMR groups was lower than the 3-mm CMR groups with the same post and core system and control group. To the best of the authors' knowledge, no similar study is available on the effect of length of the relocated margin in CMR on fracture resistance. Nonetheless, it should be noted that the resistance of sound tooth structure supporting the core against the applied forces decreases in 6-mm CMR groups due to reduction in dimensions of the residual tooth structure and increased dimensions of the reconstructed core and the resultant reduction in the integrity of the tooth structure. Also, increased dimensions of the core material increase the difference in plastic deformation of sound tooth structure and post and core system in response to mechanical loading [24]. Therefore, the reduction in fracture resistance in the 6-mm CMR groups, compared with 3 mm, can be justified.

In the 6-mm CMR groups, PFP yielded higher fracture resistance than CP. However, no significant difference was found in comparison of other groups. Considering the larger dimensions of the reconstructed core in the 6-mm CMR groups and more prominent role of core in fracture resistance against the applied forces in these groups, higher fracture resistance of the PFP and PEFRC groups can be attributed to a modulus of elasticity closer to that of tooth structure, and more uniform distribution of forces, compared with CP [41]. On the other hand, it may be stated that higher adhesion to dental substrate reinforces the residual tooth structure and yields higher fracture resistance [41]. The results of previous studies in this regard have been conflicting. Some studies reported no or insignificant effect of the applied techniques in the PEFRC groups on fracture resistance [27, 42]. In comparison of CP and PFP, some studies [43, 44] reported their comparable fracture resistance, while some others reported higher fracture resistance of CP [30]. Since none of the previous studies used the CMR approach in tooth preparation, precise comparison of the present results with the findings of previous studies is not possible.

In assessment of the mode of failure, CP showed the lowest frequency of favorable fractures. Similar results were reported by previous studies [7, 30, 43]. Comparison of the 3-mm and 6-mm CMR groups with the same post and core system revealed higher frequency of favorable fractures in the 6-mm CMR group. This finding can be explained by greater destruction of coronal structure and its fracture prior to load transfer to the apical region. Unlike the PFP, PEFRC, and CP-6 groups in which favorable fractures had the highest frequency, unfavorable fractures were more common in the CP-3 group, which can be probably due to the higher modulus of elasticity of CP along with greater amount of residual coronal structure in this group in comparison with CP-6, and the resultant apical transfer of the applied forces prior to fracture of the coronal structure.

This study had some limitations. Despite applying strict inclusion and exclusion criteria for maximum standardization of teeth, the collected teeth did not have the same age due to limited availability of extracted teeth. Evidence shows that the teeth in older individuals are more susceptible to fracture than in younger individuals [45]. Nonetheless, due to this matter, the obtained results are not limited to a specific age group and have higher generalizability although a larger sample size would have been ideal. Since the present study is among the very first to address this topic, future studies are recommended to address the abovementioned limitations. Moreover, this study had an in vitro design. Thus, complete simulation of the oral environment was not possible. Also, the results cannot be generalized to other restoration types, tooth types, and different patterns of destruction of tooth structure. Therefore, future studies are recommended to assess the correlation of length of the relocated margin and pattern of destruction in CMR and their effect on fracture resistance and marginal adaptation, compared with other methods of accessing the subgingival margin. Clinical studies in this respect are also warranted.

## 5. Conclusion

Thermomechanical loading significantly decreased the marginal integrity at all interfaces. In the 3-mm CMR groups, the highest marginal integrity was noted in the PEFRC group, followed by the PFP and CP groups. In the 6-mm CMR groups, the marginal integrity in the CP group was lower than in the PFP and PEFRC groups at the luting cement-core interface and lower than that in the PEFRC group at the core-tooth interface. CP had higher fracture resistance than PFP, but the PEFRC group had no significant difference with CP and PFP in this regard. The marginal integrity and fracture resistance decreased by an increase in length of the relocated margin in the CMR groups, but the frequency of favorable fractures also increased compared with the 3-mm CMR groups with the same post and core system.

## Figures and Tables

**Table 1:** Description of the experimental groups.

Groups	Post and core system	Length of relocated cervical margin (mm)
PFP-3	Prefabricated fiber post and composite core	3
PEFRC-3	Polyethylene fiber-reinforced composite	3
CP-3	Casting post and core	3
PFP-6	Prefabricated fiber post and composite core	6
PEFRC-6	Polyethylene fiber-reinforced composite	6
CP-6	Casting post and core	6
Control	No preparation	

**Table 2:** Marginal integrity at the crown-luting cement interface in all test groups in percentage (*n* = 10).

Groups	PFP-3	PFP-6	PEFRC-3	PEFRC-6	CP-3	CP-6	*P* value (statistic)
Before TML mean ± SD	98.70 ± 0.90	98.19 ± 1.40	98.20 ± 1.12	98.11 ± 1.08	98.33 ± 1.48	99.04 ± 1.23	0.382 (5.29) ^*∗*^
After TML mean ± SD	85.09 ± 1.94	84.93 ± 2.85	98.20 ± 1.12	85.09 ± 1.63	85.01 ± 2.19	85.05 ± 1.94	0.999 (0.03)^†^
*P* value (statistic)	<0.001 (19.21)^‡^	<0.001 (12.24)^‡^	<0.001 (25.63)^‡^	<0.001 (23.05)^‡^	<0.001 (15.70)^‡^	0.008 (2.67)^††^	—

Level of significance, 0.05; PFP, prefabricated fiber post; PEFRC, polyethylene fiber-reinforced composite; CP, casting post; 3 and 6, length of relocated cervical margin in millimeter; SD, standard deviation.  ^*∗*^Kruskal–Wallis. ^†^ANOVA. ^‡^Paired sample test. ^††^Wilcoxon signed ranks.

**Table 3:** Marginal integrity at the luting cement-core (LC-Co), core-teeth (Co-T), and luting cement-enamel (LC-E) interfaces in all test groups in percentage (*n* = 10).

Groups		PFP-3	PEFRC-3	CP-3	LC-E	*P* value (statistic)	PFP-6	PEFRC-6	CP-6	LC-E	*P* value (statistic)
Before TML	LC-Co	95.20 ± 1.57^Aa^	96.18 ± 1.69^Aa^	85.20 ± 0.83^Ba^	99.11 ± 0.83^C^	<0.001 (196.89) ^*∗*^	94.53 ± 1.08^Aa^	97.35 ± 1.04^Ba^	83.25 ± 1.59^Ca^	99.11 ± 0.83^D^	<0.001 (334.78) ^*∗*^
	CO-T	95.38 ± 3.31^Aa^	98.15 ± 1.64^Ab^	63.28 ± 2.88^Cb^	99.11 ± 0.83^B^	<0.001 (470.26)^†^	93.27 ± 2.70^Aa^	94.11 ± 3.66^Aa^	50.40 ± 2.45^Bb^	99.11 ± 0.8^C^	<0.001 (676.38) ^*∗*^
	LC-E	99.11 ± 0.83^b^	99.11 ± 0.83^b^	99.11 ± 0.83^c^	99.11 ± 0.83	—	99.11 ± 0.83^b^	99.11 ± 0.83^b^	99.11 ± 0.83^c^	99.11 ± 0.83	—
	*P* value (statistic)	0.001 (9.30)^†^	<0.001 (9.65) ^*∗*^	<0.001 (909/52)^†^	—	—	0.001 (27.83) ^*∗*^	0.001 (11.44)^†^	<0.001 (1802/94) ^*∗*^	—	—

After TML	LC-Co	78.21 ± 1.55^Aa^	85.07 ± 1.92^Ba^	60.85 ± 4.33^Ca^	89.22 ± 2.77^D^	<0.001 (173.26) ^*∗*^	70.21 ± 3.64^Aa^	74.05 ± 1.63^Aa^	50.39 ± 5.75 ^Ba^	89.22 ± 2.77^C^	<0.001 (162.18)^†^
	CO-T	75.18 ± 3.78^Aa^	3.55 ^Bb^ ± 88.44	59.13 ± 2.56^Ca^	89.22 ± 2.77^B^	<0.001 (174.78) ^*∗*^	66.69 ± 3.86^Aba^	71.12 ± 2.15^ACb^	44.70 ± 2.15 ^Bb^	89.22 ± 2.77^C^	<0.001 (31.30)^‡^
	LC-E	99.11 ± 0.83^b^	99.11 ± 0.83^b^	99.11 ± 0.83^b^	99.11 ± 0.83	—	99.11 ± 0.83^b^	99.11 ± 0.83^c^	99.11 ± 0.83^c^	99.11 ± 0.83	—
	*P* value (statistic)	<0.001 (60.45) ^*∗*^	0.011 (5.49) ^*∗*^	0.001 (233.75) ^*∗*^	—	—	0.001 (18.32)^‡^	<0.001 (170.30) ^*∗*^	<0.001 (349.22)^†^	—	—

PFP, prefabricated fiber post; PEFRC, polyethylene fiber-reinforced composite; CP, casting post; 3 and 6, length of relocated cervical margin in millimeter. Values with the same uppercase letters in the same row are not significantly different (*P*  > 0.05). Those with different uppercase letters are significantly different (*P*  < 0.05). Values with the same lowercase letters in the same column are not significantly different (*P*  > 0.05). Those with different uppercase letters are significantly different (*P*  < 0.05).  ^*∗*^ANOVA with post hoc Tukey test. ^†^ANOVA with post hoc Games–Howell test. ^‡^Kruskal–Wallis with Bonferroni correction test.

**Table 4:** Fracture resistance of the groups in Newtons (*n* = 10).

Groups	PFP	PEFRC	CP	CTR	*P* value (statistic)
3 mm	2,742.15 ± 494.57^Aa^	2,881.63 ± 466.76^Aa^	3,073.85 ± 292.24^Aa^	2,948.13 ± 177.76^A^	0.614 (1/81) ^*∗*^
6 mm	1,799.54 ± 104.66^Ab^	1,533.70 ± 261.45^Ab^	1,481.95 ± 148.38^Bb^	2,948.13 ± 177.76^C^	<0.001 (127.38)^†^
Control	2,948.13 ± 177.76^a^	2,948.13 ± 177.76^a^	2,948.13 ± 177.76^a^	—	—
*P* value (statistic)	<0.001 (35.26)^‡^	<0.001 (54.11)^†^	<0.001 (17.56)^††^	—	—

PFP, prefabricated fiber post; PEFRC, polyethylene fiber-reinforced composite; CP, casting post. Values with the same uppercase letters in the same column are not significantly different (*P*  > 0.05). Those with different uppercase letters are significantly different (*P*  < 0.05). Values with the same lowercase letters in the same column are not significantly different (*P*  > 0.05). Those with different uppercase letters are significantly different (*P*  < 0.05). ^*∗*^Kruskal–Wallis. ^†^ANOVA with post hoc Games–Howell. ^‡^ANOVA with post hoc Tukey. ^††^Kruskal–Wallis with Bonferroni correction.

**Table 5:** Comparison of failure modes of the 10 groups by the Fisher–Freeman–Halton test (*n* = 10).

Groups	Failure pattern		*P* value (statistic)
	Favorable *n* (%)	Unfavorable *n* (%)	
PFP-3	7 (77.8)	2 (22.2)	0.013 (13.31)
PFP-6	9 (100)	0 (0)	
PEFRC-3	7 (77.8)	2 (22.2)	
PEFRC-6	9 (100)	0 (0)	
CP-3	4 (44.4)	5 (55.6)	
CP-6	6 (66.7)	3 (33.3)	
CTR	9 (100)	0 (0)	

PFP, prefabricated fiber post; PEFRC, polyethylene fiber-reinforced composite; CP, casting post; CTR, control.

## Data Availability

The datasets used and/or analyzed during the current study are available from the corresponding author on reasonable request.
